# Cancer: A disease at the crossroads of trade‐offs

**DOI:** 10.1111/eva.12444

**Published:** 2016-12-26

**Authors:** Camille Jacqueline, Peter A. Biro, Christa Beckmann, Anders Pape Moller, François Renaud, Gabriele Sorci, Aurélie Tasiemski, Beata Ujvari, Frédéric Thomas

**Affiliations:** ^1^ CREECMontpellier Cedex 5France; ^2^ MIVEGECUMR IRD/CNRS/UM 5290Montpellier Cedex 5France; ^3^ Centre for Integrative EcologySchool of Life and Environmental SciencesDeakin UniversityWaurn PondsVICAustralia; ^4^ Ecologie Systématique EvolutionUniversité Paris‐SudCNRSAgroParisTechUniversité Paris‐Saclay, F‐91405 Orsay CedexFrance; ^5^ BiogéoSciencesCNRS UMR 6282Université de BourgogneDijonFrance; ^6^ Unité d'EvolutionEcologie et Paléontologie (EEP) Université de Lille 1 CNRS UMR 8198groupe d'Ecoimmunologie des AnnélidesVilleneuve‐d'AscqFrance

**Keywords:** cancer, life‐history traits, natural selection, trade‐off

## Abstract

Central to evolutionary theory is the idea that living organisms face phenotypic and/or genetic trade‐offs when allocating resources to competing life‐history demands, such as growth, survival, and reproduction. These trade‐offs are increasingly considered to be crucial to further our understanding of cancer. First, evidences suggest that neoplastic cells, as any living entities subject to natural selection, are governed by trade‐offs such as between survival and proliferation. Second, selection might also have shaped trade‐offs at the organismal level, especially regarding protective mechanisms against cancer. Cancer can also emerge as a consequence of additional trade‐offs in organisms (e.g., eco‐immunological trade‐offs). Here, we review the wide range of trade‐offs that occur at different scales and their relevance for understanding cancer dynamics. We also discuss how acknowledging these phenomena, in light of human evolutionary history, may suggest new guidelines for preventive and therapeutic strategies.

## 1 Introduction

Although medical and evolutionary sciences have traditionally developed in relative isolation (Williams & Nesse, 1991), it is widely acknowledged that cancer is a process that is shaped by Darwinian evolution (Aktipis & Nesse, 2013; Thomas et al. 2013). Specifically, cancer is driven by both the somatic evolution of cell lineages that have escaped controls on replication, and the evolution of genes that influence cancer risk in populations (Aktipis & Nesse, 2013; Thomas et al., 2013). Therefore, applying evolutionary theories to cancer is relevant for understanding many aspects of this pathology, from its origin *per se,* to possible ways to control its progression, and how to prevent therapeutic failures (Aktipis & Nesse, 2013; Rozhok & DeGregori, 2015). Although these ideas first originated in the mid‐seventies (*e.g*., Cairns, 1975; Nowell, 1976), many promising opportunities for the application of evolutionary biology to carcinogenesis and oncology remain unexplored (Thomas et al., 2013).

Central to evolutionary theory is the assumption that living organisms are, at some level, resource‐limited and thus must face trade‐offs among competing energy demands and life‐history traits, such as development, survival, and reproduction (Roff, 1993; Stearns, 1992). Life‐history theory proposes that these trade‐offs help to determine the evolution of phenotypes and could explain the diversity of life‐history patterns found in biological populations (Hawkins, 2004). This concept has led to the idea that mammalian populations can be placed along a fast–slow continuum and has been confirmed by some empirical evidence (see Oli, 2004 for review). Species selected to mature early have high reproductive rates and short generation times and are considered to have a “fast” strategy, while species that have the opposite suite of traits are thought to represent the “slow” end of the continuum (Promislow & Harvey, 1990; Read & Harvey, 1989).

The main reason why trade‐offs have played a prominent role in evolutionary thinking is their direct link with processes limiting the adaptive potential of organisms. Trade‐offs lead to antagonistic relationships between phenotypic traits and are thought to be determined both genetically and environmentally (Box 1). A large diversity of trade‐offs have so far been identified, for example, the classical trade‐offs between reproduction and survival, offspring number and quality, and current versus future reproduction (Agrawal, Conner, & Rasmann, 2010; Stearns, 1989), or in the context of host–pathogen interactions, between immune defenses versus reproduction or between resistance versus tolerance to pathogens (Råberg, Graham, & Read, 2009; Sorci, Boulinier, Gauthier‐Clerc, & Faivre, 2008).

Box 1 **Levels of analysis**

1 Trade‐offs exist at different levels, namely phenotypic, genotypic, and at intermediate structures, which lie between the other two (Stearns, 1989). The phenotypic level concerns whole‐organism studies of traits directly connected with ontogeny, reproduction, and survival. For instance, organisms acquire limited resources and then differentially allocate them to competing functions such as growth, survival, and reproduction. The genotypic level refers to all types of evidence claimed to be genetic (using quantitative, Mendelian, or molecular genetics approaches). This evolutionary level reflects the constraints (negative genetic correlations) operating on multiple traits that are linked in ways that prevent simultaneous optimization of all of them. By intermediate structure, we imply all mechanisms connecting the genotypic to the phenotypic levels, including physiological and developmental mechanisms under endocrinological control that result in the allocation of resources among the functions of maintenance, growth, storage, reproduction, and survival (Stearns, 1989). Finally, two kinds of trade‐offs are recognized, those for which opposing selection across different environments results in a polymorphic trait and trade‐offs which are the consequences of competition for shared limiting resources and that act on multiple traits (Agrawal et al., 2010).

Similar to other pathologies, the concept of trade‐offs is increasingly considered to be central in both fundamental and applied research on cancer (e.g., Aktipis, Boddy, Gatenby, Brown, & Maley, 2013; Roche & Thomas, 2016). For instance, the emergence of malignant cell lines may be both cause and consequence of evolutionary trade‐offs. In addition, most protective mechanisms shaped by selection against cancer are costly and come with trade‐offs (Hochberg, Thomas, Assenat, & Hibner, 2013); thus, cancer can emerge as a consequence of trade‐offs with life‐history traits such as reproduction (Aktipis & Nesse, 2013).

Here, we review how taking into account these trades‐offs, from the perspective of the cancerous cells and from the perspective of the host, can help us to understand cancer dynamics. With such evolutionary approach, we suggest that it may be possible to identify aspects of anticancer adaptations that have been constrained by trade‐offs and so may be effective targets for medical interventions.

## 2 Life‐history trade‐offs at the malignant cell level

**Figure 1:**
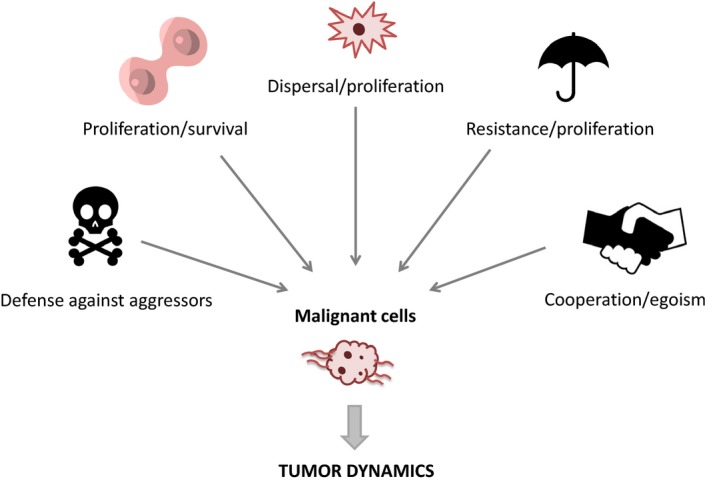
Tumor dynamics rely on numerous trade‐offs at the malignant cell level. Neoplastic cells have to face numerous trade‐offs to increase their fitness in the host

Cells become malignant by acquiring genetic mutations that lead to increased survival and reproduction. However, cancer cells are not Darwinian demons able to maximize all fitness components simultaneously; instead, recent evidence suggests that their functioning is constrained by a number of underlying trades‐offs which are summarized in Figure 1.

### 2.1 Proliferation and survival

Because malignancies develop in environments that are expected to have limited resources (e.g., space and nutrients) (Alfarouk, Ibrahim, Gatenby, & Brown, 2013), a first trade‐off in malignant cells may be between cell proliferation and survival. It has been predicted that, in a “rich” environment with high cell density, malignant cells will adopt a “fast” life‐history with rapid proliferation but low resistance to apoptosis, whereas in adverse environments, cells will have a “slow” life‐history, with low proliferation but increased survival (Aktipis et al., 2013; Alfarouk et al., 2013). Cancer cells can indeed be categorized as proliferation‐promoting or survival‐promoting phenotypes, suggesting that they can display phenotypes “adapted” to their environment (Aktipis et al., 2013). Selection for different life‐history strategies in malignant cells could also fluctuate over time because tumorigenesis and/or therapies will generate distinct selective landscapes. Aktipis et al. (2013) hypothesized that these trade‐offs may only happen in later stages of tumorigenesis, favoring extreme phenotypes such as rapidly proliferating cells with poor survival abilities or apparently dormant cells that can survive extremely well.

### 2.2 Dispersal and cell plasticity

Many animals disperse to reduce intraspecific competition (Fahrig, 2007). Models in evolutionary ecology have also shown that organisms should move only when the costs of “staying at home” outweigh the potential costs of leaving the natal habitat (North, Cornell, & Ovaskainen, 2011). Cancer cells within organs face the same trade‐off (Lee, Silva, Li, & Slifker, 2011; Marusyk & Polyak, 2010), and they can switch between migratory and proliferative phenotypes (e.g., squamous cell carcinoma; Biddle et al., 2011). A theoretical study has proposed that neoplastic cells, with deregulated metabolism, may be selected to have higher motility because their survival depends on their ability to move from a resource‐restricted region to a new environment that can sustain their high metabolic needs (Aktipis, Maley, & Pepper, 2012). Moreover, numerous evidences suggested that when malignant cells are exposed to poor “environmental conditions” (hypoxia, toxins, etc.) or when resources are spatially or temporally heterogeneous (oxygen, nutrients, etc.), selection might favor metastatic cancer phenotypes (Anderson et al., 2006; Chen, Sprouffske, Huang, & Maley, 2011; Daoust et al., 2013; but see Arnal et al., 2016). In addition to these theoretical studies, it has been observed in a murine lung cancer model that toxicity mediated by the chemotherapy may induce metastasis (Daenen et al., 2011).

Cell plasticity is pervasive in cancer stem cells which can be proliferative or quiescent according to factors describing the tumor microenvironment, with the potential to influence cellular metabolism (DeBerardinis, Lum, Hatzivassiliou, & Thompson, 2008). Because phenotypic plasticity in organisms has been shown to be costly (DeWitt, Sih, & Wilson, 1998), exploring the costs for malignant cells of expressing conditional life‐history strategies (e.g., cell plasticity) in different environments is also a promising direction for management of tumorigenesis (Aktipis et al., 2013).

### 2.3 Dispersal and cell plasticity

As infectious agents do, cancer cells are able to develop adaptations to resist to therapeutic drugs. Most of them relay on pumps that expulse and reduce intracellular concentrations of cytotoxic drugs such as ATP‐binding cassette (ABC) drug transporters commonly implied in multidrug resistance (MDR) (Fletcher, Haber, Henderson, & Norris, 2010). It has been observed that even if MDR lines (i.e., MCF‐7/Dox) do not show constrained proliferation or survival in glucose‐rich environment, they show a significantly decreased proliferation in glucose‐limited conditions because of their higher ATP demands (Kam et al., 2011). Strategies taking advantage of the trade‐off between proliferation and resistance have given rise to adaptive therapies. Such evolutionary approaches aim to combine the immediate cytotoxic effects of treatments with the ultimate goal of exploiting the adaptive responses of tumor cells (Gatenby, Brown et al., 2009; Gatenby, Silva et al., 2009). For example, a theoretical approach has confirmed that selection of chemosensitive cells has the potential to destroy malignant cells through competition (Maley et al., 2004). Sensitive cells may be selected using a combination of cytotoxic drugs with drugs decreasing energy production which may accentuate the cost of resistance and suppress the proliferation of drug‐resistant clones (Silva et al., 2012). Indeed, a new treatment strategy, designed in murine breast cancer model, has allowed a continuous decline in tumor size even with long intervals between treatments through exploitation of the fitness cost of resistance (Enriquez‐Navas et al., 2016).

### 2.4 Defenses against multiple aggressors

Decades of research in applied ecology have illustrated that the evolution of defenses against one natural enemy affects the individual's ability to defend against other enemies, and/or to optimally express other fitness‐related traits (Moret & Schmid‐Hempel, 2000; Sheldon & Verhulst, 1996). The presence of multiple enemies can modify the resistance against one specific enemy, with the direction of evolutionary change depending on enemy encounter rates, defense costs, and mechanistic interactions among defense mechanisms (Poitrineau, Brown, & Hochberg, 2003). Cancer cells, like organisms, have to face attack from many different biotic and abiotic aggressors, for example, immune system, oncolytic viruses, and therapies. It is now well recognized that cancer cells can evolve resistance mechanisms to both chemotherapy (Gottesman, 2002) and immunotherapy (Restifo, Smyth, & Snyder, 2016). As investment in one defense could influence the level of investment in another, it has been suggested that biological and chemical therapies could be coupled in a strategic multistep approach (De Pillis et al., 2006). In agreement with this view, evidence shows that tumor cells able to successfully adapt to an immune attack are more sensitive to the cytotoxic effects of chemotherapy (Antonia et al., 2006).

## 3 Life‐history trade‐offs at the organism level

### 3.1 Host protective mechanisms

Regarding fitness‐reducing diseases, evolutionary theory postulates that organisms should be under selective pressure to (i) avoid the source of the pathology in the first instance, (ii) then prevent its progression once sick, and (iii) finally alleviate the fitness costs if further development is unavoidable (Thomas, Guégan, & Renaud, 2009). This conceptual framework applies also to cancer and relies on trade‐offs (Ujvari et al., 2016).

#### 3.1.1 Cancer prevention

Environmental factors favoring cancer emergence and/or progression are potentially numerous, their origins being both anthropogenic (e.g., radioactivity; Møller & Mousseau, 2015) and natural (e.g., natural radiation levels, oncogenic pathogens, transmissible cancers, and secondary compounds of plants) (Ducasse et al., 2015). In this context, it is predicted that traits allowing individuals to reduce the frequency or the duration of exposure to those factors could be favored by selection as they decrease cancer risk, but will likely also entail costs (Vittecoq et al., 2015). Being choosy to avoid cancer risks (with respect to habitat, food, or partners with a contagious cancer) may entail a cost that is higher when competition is more intense. In addition, adopting a lifestyle reducing exposure to mutagenic factors may result in a small selective advantage if most cancers are due to intrinsic factors (Thomas, Roche et al. 2016; Tomasetti & Vogelstein, 2014). Thus, considering that the probability of developing an aggressive cancer before or during the reproductive period is low (Ujvari et al., 2016) and that the perception of cancer risk may be unreliable, the benefit of prevention may be offset by its cost.

Even when a given trait (e.g., behavior) strongly contributes to protect an individual from a fitness‐impacting cancer, it may not necessarily increase in frequency in the population. Indeed, when a trait is both essential to the host's survival and reproduction, but also enhances cancer risks, it will be subject to an evolutionary trade‐off. Natural selection will most likely favor individuals whose trait expression ensures the optimal compromise between satisfying a need (e.g., reproduction) and minimizing the risk of detrimental consequences on health (Vittecoq et al., 2015). For instance, in the case of Tasmanian devil (*Sarcophilus harrisii*) and their contagious cancer (Murchison, 2009; Pye et al., 2016), natural selection cannot strongly favor individuals avoiding conspecifics, because at the same time, sexual selection favors individuals displaying aggressive biting behavior that increases mating and breeding success (Ujvari et al., 2016).

Thus, despite the abundance of ecological contexts that are associated with cancer risks, there are only a few demonstrated examples of behavioral adjustments in wildlife species related to cancer prevention (e.g., antioxidant consumption; Senar et al., 2010). Acknowledging that most, if not all, ecosystems on our planet are now polluted by mutagenic substances to a greater extent than ever before (Ducasse et al., 2015), it is predicted that natural selection will favor cancer prevention to avoid fitness loss. Thus, for numerous species, the benefits of behavioral adjustments will surpass their costs and examples of prevention could substantially increase in the future.

#### 3.1.2 Cancer suppression

Cancer appeared at the dawn of multicellularity (i.e., more than half a billion years ago (Merlo, Pepper, Reid, & Maley, 2006; Nunney, 2013)), and a major aspect of this transition from unicellular ancestors has been the suppression of cell‐level fitness to promote organism‐level fitness (Maynard‐Smith & Szathmary, 1997; Michod, 2000). Effective multicellularity, therefore, required both cell cooperation and mechanisms for eliminating conflicts arising from mutations that can enhance cell‐level fitness at the expense of the individual organism (Aktipis et al., 2015; Michod & Roze, 2001). This situation resulted in selective pressures favoring the evolution of many cancer suppression mechanisms, from cell‐intrinsic checks that prevent cellular proliferation and invasion of other tissues and organs, to integral controls that suppress cancer by operating at the level of tissue organization. These mechanisms include, but are not limited to, apoptosis, effective DNA repair, epigenetic modifications, cell cycle checkpoints, telomere shortening, tissue architecture, and immune surveillance (DeGregori, 2011).

As evolution shaped larger and longer lived multicellular organisms, the problem of cancer suppression became even more challenging (Nunney, 2013; Peto, Roe, Lee, Levy, & Clack, 1975). To counteract the increased risk of developing a cancer, large and long‐lived species may have optimized their tumor suppression mechanisms and adopted lower somatic mutation rates, redundancy of tumor suppressor genes, etc. (Abegglen et al., 2015; Caulin & Maley, 2011). However, solutions for suppressing cancer are not perfect, even if they are remarkably effective. For example, tumor suppressor genes and their resulting products sometimes fail to repair double‐stranded DNA breaks without introducing errors (Khanna & Jackson, 2001). Multicellular organisms could theoretically have better mechanisms to suppress cancer, but the costs of such mechanisms might exceed the benefits when individuals reach a postreproductive age (Hochberg et al., 2013). Thus, natural selection has mainly favored the evolution of anticancer adaptations that act before or during reproductive age (Crespi and Summers^2005^; DeGregori, 2011). Another way to manage the cost of cancer suppression mechanisms could be to select protective “low‐cost” mechanisms that do not involve complete tumor elimination. For instance, auto‐immunity is costly for organisms and immune tolerance occurs in response both to infections (Read, Graham, & Råberg, 2008) and cancer (Mapara & Sykes, 2004). Even if it precludes the development of an adequate antitumor response, tolerance might be a better strategy than a costly overactivation of the immune system. Interestingly, strategies for breaking this tolerance have been studied as therapeutic approaches and led to promising results (Makkouk & Weiner, 2015). For instance, the use of immune checkpoint blockades, that abrogate negative signals diminishing T‐cell activation during the priming process, has been approved for the treatment of advanced melanoma (Ipilimumab) (Page et al., 2012).

A recent study argues that adopting a more organ‐centered approach is desirable for a full understanding of organ‐specific trade‐offs associated with cancer suppression mechanisms (Thomas, Roche et al., 2016). Organs are the products of adaptation to natural and sexual selection (Nesse & Williams, 1996). The importance and/role of various organs in keeping the organism functional and ultimately to maximize fitness vary substantially. For instance, organs such as heart, brain, or pancreas are absolutely essential for survival, while others such as gallbladder are disposable. Organ‐specific trade‐offs have already been suggested in association with the energy allocated to their development. As some organs are considered to be metabolically expensive such as brain, gut, and sexual organs, the expensive‐tissue hypothesis suggests that the allocation of energy to develop large brains results in a relatively smaller gut (Aiello, Wheeler, & Wheeler, 1995). Other studies have proposed an expensive sexual tissue hypothesis that relies on negative relationship between investment in testes and investment in brain (Pitnick, Jones, & Wilkinson, 2006). In the context of cancer suppression, organ protection may not be maximal but rather suboptimal and unequal due to trade‐offs with the other organs and mechanisms, preventing malignant transformation *per se*, should be more prominent in vital organs in order to preserve their functionality (Thomas, Nesse et al., 2016).

Finally, suppression of neoplastic growths may lead to trade‐offs in other essential functions that require cell proliferation (Aktipis & Nesse, 2013). First, the capacity to repair tissues while limiting uncontrolled cell division represents a key trade‐off for any multicellular organism (Aktipis & Nesse, 2013). Second, cell capacities during embryogenesis and development (e.g., “invasion” of cells into other developing tissues during gastrulation), which are essential for reproducing organisms, confer a risk for developing cancer (Ben‐David & Benvenisty, 2011). Finally, the p53 protein, with a well‐known cancer‐suppressive function, leads to reduced tissue renewal and repair, stem cell deletion, and organismal aging through an antagonistic pleiotropy effect (Campisi, 2002, 2003; García‐Cao et al., 2002). For instance, mice carrying a *p53* mutation (with a phenotypic effect analogous to the upregulation of the gene) have a lower risk of cancer development, but their life span is reduced and accompanied by early tissue atrophy (Donehower, 2002; Tyner et al., 2002). However, a recent study contradicts this observation in mice and has demonstrated that elephants, that are long‐lived mammals, have a high number of extra p53 copies (Abegglen et al., 2015). Thus, studying how elephants have overstepped the trade‐off that governs cancer suppression by p53 is of crucial interest to design therapies that reduce the cost of natural mechanisms and thus indirectly improve cancer suppression.

#### 3.1.3 Alleviating the fitness consequences of cancer

There are several examples in the parasitological literature illustrating that hosts, unable to resist infection by other means (e.g., immunological resistance, inducible defenses, or long‐distance migration), present adaptive and flexible life‐history traits that partly compensate parasite‐induced fitness reduction (e.g., by reproducing earlier; Forbes, 1993; Hochberg, Michalakis, & de Meeus, 1992; Michalakis & Hochberg, 1994). Although further evidences would be welcome, similar life‐history trait adjustments may also exist in hosts harboring tumors (Ujvari et al., 2016). From an epidemiological point of view, such responses could contribute to influence cancer risks through the evolution of differential cancer vulnerabilities. For instance, BRCA1 and BRCA2 mutations are inherited and predispose women to breast and ovarian cancer, but even though carriers of these mutations have reduced survival, they also have enhanced fertility (Easton, Ford, & Bishop, 1995; Smith, Hanson, Mineau, & Buys, 2012). Similarly, the shorter CAG repeat region within the androgen receptor gene increases an individual's risk of developing prostate cancer, but it also increases fertility earlier in life (Summers & Crespi, 2008). These findings may indicate an adaptive response to compensate the risk of fitness loss due to cancer predisposition. Such adaptive response is of considerable significance as it may allow the persistence of mutations with deleterious effects across generations (Vittecoq et al., 2015). Thus, we suggest that the existence of life‐history trait adjustments could influence the persistence of oncogenic mutations over evolutionary time (Ujvari et al., 2016).

### 3.2 Costs of reproduction

Evolution produces biological entities that tend to increase reproductive success, sometimes at the cost of health and longevity. In fact, the presence of early‐/late‐life trade‐offs has been supported and suggests that individuals have to trade somatic maintenance later in life for high allocation to reproduction early in life because of resource‐limited environments (Lemaitre et al., 2015). Costs of reproduction are therefore fundamental to understand diseases, including cancer. Mechanisms underlying the cost of reproduction are numerous from hormonal regulation to reduced immune functioning and lower defenses against stress and toxicity (Harshman & Zera, 2007). This suggests that selection for increased reproduction could also result in increased cancer susceptibility.

#### 3.2.1 Sexual competition

There is little evidence on the effect of intrasexual selection (e.g., male–male agonistic interactions) on cancer risk. In the case of a very specific type of cancer, the transmissible Tasmanian devil tumor disease, it has been reported that Tasmanian devils have higher risk of contracting cancer when they compete with other males for mates (Hamede, Bashford, McCallum, & Jones, 2009; Pearse & Swift, 2006).

In the context of intersexual selection, secondary sexual traits (e.g., impressive ornaments) may also select for mechanisms that enable rapid cell proliferation that could, in turn, enhance tumor formation as suggested by a theoretical study (Boddy et al., 2015). In addition, enhanced allocation of energy to secondary sexual traits rather than to mechanisms of somatic maintenance (DNA repair or immune defenses) could also elevate cancer risk by increasing accumulation of somatic mutations (Boddy et al., 2015). Knowing that success in mating competition has greater reproductive payoffs for males than for females, investment in competitive abilities at the expense of cancer risks should be particularly prominent in males. Antagonistic pleiotropy might also favor the persistence of mutations that confer a reproductive benefit to the carrier even if they increase the cancer risk of an individual. For example, in *Xiphophorus* fish, melanoma‐promoting oncogene alleles are associated with larger body size and aggressiveness and confer early‐life advantages in male–male competition and female mate choice (Fernandez & Bowser, 2010; Fernandez & Morris, 2008). One important limitation to studying the link between sexual competition and cancer risk is methodological constraints. Generally, investigations of organisms in the wild do not test living individuals for the presence of tumors and the assessment of the cause of death is often impossible. Development of noninvasive methods to detect cancer as well as population monitoring is crucial to progress our understanding of the impact of trade‐offs between reproduction and cancer risk.

#### 3.2.2 Sexual hormones

Estrogens are the principal hormones that regulate the female reproductive cycle and have been particularly associated with receptive behaviors (Lynch, Rand, Ryan, & Wilczynski, 2005). Thus, females producing high levels of estrogens could be selected in a context of intrasexual competition. Recent studies have highlighted the strong interactions between estrogens and immune system activation (see Khan & Ansar Ahmed, 2016 for review). High levels of estrogens during pregnancies could increase cancer cell elimination and could explain the negative correlation between parity and risk for several cancers (Chen, Gong, & Wu, 2016; Wu et al., 2015). Nevertheless, evidence suggests that circulating estrogens are associated with increased postmenopausal breast cancer risk (Key, Appleby, Barnes, Reeaves, & Al, 2002), suggesting antagonist pleiotropy even if mechanisms are not well identified. Finally, modification of modern reproductive patterns has been associated with an increase in estrogen‐positive receptor (ER+) breast cancer suggesting that women experience the cost of a modernity mismatch (Aktipis, Ellis, Nishimura, & Hiatt, 2014).

For males, testosterone has been reported to have an immunomodulatory role by reducing cytokine responses to infection (Trumble et al., 2016). Regarding cancer development, Alvarado (2013) provided evidence that in human subpopulations where competition is intense (e.g., polygamous societies), males have higher testosterone levels and increased mating success, but at the cost of increased rates of prostate cancer. However, recent studies do not report significant association between testosterone concentrations and prostate cancer (Roddam, Allen, Appleby, & Key, 2008). Nevertheless, considering hormones involved in reproductive behaviors could be of interest in social species with hierarchical dominance. In fact, subordinate individuals show a high level of stress associated with low levels of circulating testosterone, but also with a suppressed immune system through production of glucocorticoids (Sapolsky, 2016). Social species, with differential cancer risk between dominant and subordinate individuals, could be a relevant biological model to study interactions between sexual hormones, immune system, and cancer development.

### 3.3 Eco‐immunological trade‐offs

#### 3.3.1 Infectious disease burdens

Throughout evolutionary history, humans have been exposed to a large number of diverse infectious agents (Wolfe, Dunavan, & Diamond, 2007). In wealthy countries, the decreased prevalence of infectious diseases, in particular of those caused by helminths, has been paralleled by an increased incidence of cancers (Zacharia, Zacharia, & Sherman, 2003). The helper T lymphocytes may have a central role for understanding the link between infections and cancer dynamics. In fact, several studies have shown that Th1 response is protective against several cancers (Haabeth et al., 2011; Ingels et al., 2014) and intracellular pathogens. In contrast, Th2 activation is linked to negative prognosis in some cancers (Lippitz, 2013) and it confers protection against macroparasites. A trade‐off may exist because the cytokines that instruct immune cells to differentiate into the Th1 pathway tend to inhibit Th2 effectors (Kidd, 2003). As selection may have favored one specific pathway at the expense of the other in our ancestral environment, the rapid and radical change of our infectious environment may create a mismatch with consequences for cancer risk (Oikonomopoulou et al., 2013). One example of such a genetic trade‐off comes from the relative vulnerability of African Americans to malignant diseases compared with Caucasian Americans (Walker, Figgs, & Zahm, 1995). Relocation of Africans from tropical countries, where inflammation following Th2 activation was beneficial, to North America, and the consequent decrease in infection risk, may have exposed them to a higher risk of cancer (O'Byrne & Dalgleish, 2000). The use of immunoregulatory helminth products has been suggested to reduce inappropriate pro‐inflammatory responses and thereby cancer risk (Finlay, Walsh, & Mills, 2014).

#### 3.3.2 Sleep

Another trade‐off may exist between developing a powerful immunological memory to eradicate cancer by sleeping longer and short‐term survival by prospecting for food and limiting predation risk. In fact, by avoiding long periods of sleep, species reduce their risk of being predated (Lima, Rattenborg, Lesku, & Amlaner, 2005). Humans are not an exception, and it has been proposed that human sleep has been selected to be deeper but significantly shorter than for other primate species (Samson & Nunn, 2015). However, it has been shown that sleep has a specific role in the formation of immunological memory (Besedovsky, Lange, & Born, 2012). A link between cancer risk and sleep has been identified in a cohort of women with breast cancer, where it has been reported that women who routinely sleep few hours may develop more aggressive breast cancers compared with women who sleep long hours (Thompson & Li, 2013).

#### 3.3.3 Growth

At the species level, immunity could trade‐off against growth. Species with a long embryonic period have been hypothesized to invest more energy to develop an immune system that is able to resist to infections (Ricklefs, 1992). Thus, because species with higher growth rates will be those with higher rates of cell division and weaker immune systems, they should also be those with higher incidence of tumors (van der Most, de Jong, Parmentier, & Verhulst, 2011).

## 4 Concluding Remarks and Perspectives

**Figure 2:**
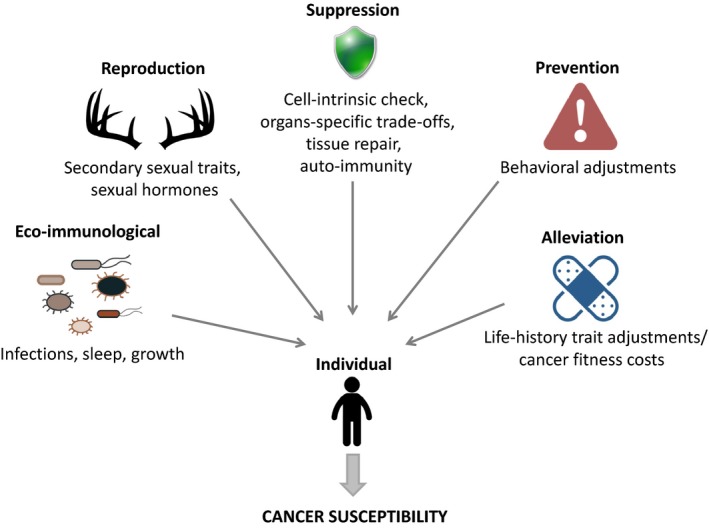
Cancer susceptibility rely on numerous trade‐offs at the individual level. Most of the protective mechanisms of the host are retained by selection against cancer and rely on underlying trade‐offs. Host susceptibility to cancer can also emerge as a consequence of other trade‐offs in organisms

There are some compelling indications that cancer dynamics may rely on and perhaps originate from numerous trade‐offs at the malignant cell and the organismal level (i.e., ontogenetic, physiological, and anatomical scales summarized in Figure 2). Acknowledging it not only permits us to highlight fundamental processes governing cancer, but also allows us identifying tools that could be useful in the development of new therapeutics.

At the cellular level, future studies taking an evolutionary–ecological perspective into account are needed to uncover novel adaptations of malignant cells and elucidate the trade‐offs under which they have evolved. Such knowledge could lead to therapies that favor cancer cells with slow life‐history strategies (high survival, low dispersal, and slow development), thereby allowing for long‐term cancer control (Aktipis et al., 2013). Emerging disciplines such as evolutionary cell biology (Lynch et al., 2014) are promising for understanding the diversity of trade‐offs governing the functioning of malignant cells, their consequences for tumor evolution, and their implications for novel therapies. For example, certain malignant cells may behave as social parasitic clones within the tumor benefiting from the metabolic investment made by their neighbors (e.g., stimulating neo‐angiogenesis and the release of growth factors) without experiencing costs themselves (Merlo et al., 2006). Interestingly, it has been proposed that these “cheaters” may create a hypertumor, which could in return damage or destroy the original neoplasm (Nagy et al., 2007). However, the existence of trade‐offs at the cell level (and hence their potential use for therapies) implicitly suggests that resources are limited. At the moment, the extent to which this is the case must be clarified because, for instance, malignant cells could manipulate their host to obtain higher level of resources (Tissot et al., 2016).

By harnessing modern technology as a supplement to natural anticancer adaptations acquired through natural selection, medical intervention could modify trade‐offs providing opportunities for prevention, detection, and treatment that have not previously been available to humans (Table 1). For example, we already employ agents like nonsteroidal anti‐inflammatory drugs to prevent malignant cancer cell growth (Rothwell et al., 2012), acting through the reduction of an inflammatory response that is nonoptimal in a modern environment. In addition, identification of trade‐offs involving behavioral habits can lead to prevention, such as condom use (to prevent transmission of cancer‐causing viruses) and increased sleeping time, which can reduce cancer risk. In a cancer elimination context, engaging the immune system into the systematic elimination of precancerous lesions via prophylactic cancer vaccination is a promising research direction (e.g., Yaddanapudi et al., 2012). However, this intervention technique becomes detrimental to individual health if it results in overly frequent (if not constant) activation of the immune system. Intervening with the body's own tumor suppressor mechanisms could unbalance system homeostasis and result in unintended consequences such as collateral tissue damage, autoimmune complications, and nonspecific side effects. Therefore, it is important to notice that modern technologies could also result in costs like overdiagnosis and overtreatment.

**Table 1:** Different scales of trade‐offs and their potential applications in public health: evidences from theoretical, experimental and clinical studies

Level	Trade‐offs	Potential interventions suggested by trade‐off framework	Theoretical references	Experimental references	Clinical references
Cellular	Proliferation/Survival	Resource limitation and normalizing therapy to favor survival	Aktipis et al., (2013)	Gatenby, Silva et al., (2009)	
	Dispersal/Proliferation	Blockage of epithelial–mesenchymal transition (EMT)	Anderson et al., (2006); Daoust et al., (2013)	Aref et al., (2013), Davis, Stewart, Thompson, & Monteith, (2014)	
	Resistance/Proliferation	Adaptive therapy	Maley et al., (2004)	Enriquez‐Navas et al., (2016)	
	Defenses against multiple aggressors	Combination of biological and chemical therapies	De Pillis et al., (2006)	Machiels et al., (2001); Wheeler, Das, Liu, Yu, & Black, (2004)	Huncharek, Caubet, & McGarry, 2001
	Cooperation/Egoism	Use hyper‐tumor to destroy original neoplasm	Marusyk et al., (2014); Nagy et al., (2007)	Archetti, Ferraro, & Christofori, (2015)	
Individual	Reproduction/Cancer	Condom use, vaccines against oncogenic pathogens			Söderlund‐Strand, Uhnoo, & Dillner, (2014)
		Preventive removal of the organ (e.g., Mastectomies for women BRCA1/2)			Meijers‐Heijboer et al., (2001)
		Hormonal treatments (e.g., post‐menopausal women and breast cancer)	Boddy et al., (2015)		Goss et al., (2011)
	Immune tolerance/Auto‐immunity	Immunotherapy (e.g., immune checkpoint inhibitors)		Beatty et al., (2011)	Page et al., (2012)
	Responses to infection/Cancer	Use of helminths regulatory products	Harnett & Harnett, (2010)	León‐Cabrera et al., (2014)	
		Anti‐inflammatory drugs	Oikonomopoulou et al., (2013)	Valle et al., (2013)	Rothwell et al., (2012)
	Sleep/Predation	[Speculative] Favor longer sleep (sleeping pills)			

Modern humans live longer than our ancestors because of benign and infrequent exposure to infectious diseases, predation, and violence. This situation results in increased cancer incidence because the largest risk factor for most cancers is simply aging (Frank, 2007). By considering differences between what natural selection acts on (reproductive fitness) and our current goals in the modern world (improving health and well‐being), it may be possible to identify aspects of anticancer adaptations that have been constrained by trade‐offs and so may be effective targets for intervention. In fact, as evolution has not apparently strongly selected for late‐life cancer prevention (Frank, 2007), this may represent an opportunity to “do better” than evolution's current prescription. For instance, ontogeny requires that cells have the capacity to move and proliferate, capacities which may be “reactivated” in cancer. After development is completed, it may be possible to constrain somatic cell phenotypes so that they are less likely to call upon developmental programs reducing risk of cancer development.

To conclude, we emphasize the benefits of playing by the evolutionary rules that regulate the risk of cancer, rather than adopting treatment of symptoms. This work calls for a more integrated approach to cancer research that will consider evolutionary trade‐offs. In particular, treatment strategies that will shift these trades‐offs away from cancer and favor the host may be a promising way to discover new methods of prevention and treatment.

## References

[eva12444-bib-0001] Abegglen, L. M. , Caulin, A. F. , Chan, A. , Lee, K. , Robinson, R. , Campbell, M. S. , … Schiffman, J. D. (2015). Potential mechanisms for cancer resistance in elephants and comparative cellular response to DNA damage in humans. JAMA, 314, 1850–1860. 26447779 10.1001/jama.2015.13134 PMC4858328

[eva12444-bib-0002] Agrawal, A. , Conner, J. , & Rasmann, S . (2010). Tradeoffs and negative correlations in evolutionary ecology.

[eva12444-bib-0003] Aiello, L. C. , Wheeler, P. , & Wheeler, P. (1995). Primate evolution the expensive‐tissue hypothesis the brain and the digestive. Current Anthropology, 36, 199–221.

[eva12444-bib-0004] Aktipis, C. A. , Boddy, A. M. , Gatenby, R. A , Brown, J. S. , & Maley, C. C. (2013). Life history trade‐offs in cancer evolution. Nature Reviews. Cancer, 13, 883–892. 24213474 10.1038/nrc3606 PMC4010142

[eva12444-bib-0005] Aktipis, C. A. , Boddy, A. M. , Jansen, G. , Hibner, U. , Hochberg, M. E. , Maley, C. C. , & Wilkinson, G. S. (2015). Cancer across the tree of life: Cooperation and cheating in multicellularity. Philosophical transactions of the Royal Society of London. Series B, Biological sciences, 370, 20140219–20140219. 26056363 10.1098/rstb.2014.0219 PMC4581024

[eva12444-bib-0006] Aktipis, C. A. , Ellis, B. J. , Nishimura, K. K. , & Hiatt, R. A. (2014). MODERN REPRODUCTIVE PATTERNS NOT ASSOCIATED WITH ER‐NEGATIVE BREAST CANCER. Evol. Med: Public Heal. 10. 10.1093/emph/eou028 PMC4362290 25389105

[eva12444-bib-0007] Aktipis, A. , Maley, C. C. , & Pepper, J. W. (2012). Dispersal evolution in neoplasms : The role of disregulated metabolism in the evolution of cell motility. Cancer Prevention Research (Philadelphia, Pa.), 5, 266–275. 10.1158/1940-6207.CAPR-11-0004 PMC3273626 21930797

[eva12444-bib-0008] Aktipis, C. A. , & Nesse, R. M. (2013). Evolutionary foundations for cancer biology. Evolutionary Applications, 6, 144–159. 23396885 10.1111/eva.12034 PMC3567479

[eva12444-bib-0009] Alfarouk, K. O. , Ibrahim, M. E. , Gatenby, R. A , & Brown, J. S. (2013). Riparian ecosystems in human cancers. Evolutionary Applications, 6, 46–53. 23396634 10.1111/eva.12015 PMC3567470

[eva12444-bib-0501] Alvarado, L. C. (2013). Do evolutionary life‐history trade‐offs influence prostate cancer risk? a review of population variation in testosterone levels and prostate cancer disparities. Evolutionary Applications, 6(1), 117–133. 23396824 10.1111/eva.12036 PMC3567477

[eva12444-bib-0010] Anderson, A. R. , Weaver, A. M. , Cummings, P. T. , & Quaranta, V. (2006). Tumor morphology and phenotypic evolution driven by selective pressure from the microenvironment. Cell, 127, 905–915. 17129778 10.1016/j.cell.2006.09.042

[eva12444-bib-0011] Antonia, S. J. , Mirza, N. , Fricke, I. , Chiappori, A. , Thompson, P. , Williams, N. , … Gabrilovich, D. I. (2006). Combination of p53 cancer vaccine with chemotherapy in patients with extensive stage small cell lung cancer. Clinical Cancer Research, 12, 878–887. 16467102 10.1158/1078-0432.CCR-05-2013

[eva12444-bib-0012] Archetti, M. , Ferraro, D. A , & Christofori, G. (2015). Heterogeneity for IGF‐II production maintained by public goods dynamics in neuroendocrine pancreatic cancer. Proceedings of the National Academy of Sciences of the United States of America, 112, 1833–8. 25624490 10.1073/pnas.1414653112 PMC4330744

[eva12444-bib-0013] Aref, A. R. , Huang, R. Y.‐J. , Yu, W. , Chua, K.‐N. , Sun, W. , Tu, T.‐Y. , … Kamm, R. D. (2013). Screening therapeutic EMT blocking agents in a three‐dimensional microenvironment. Integrative Biology : Quantitative Biosciences From Nano To Macro, 5, 381–9. 23172153 10.1039/c2ib20209c PMC4039387

[eva12444-bib-0014] Arnal, A. , Tissot, T. , Ujvari, B. , Nunney, L. , Solary, E. , Laplane, L. , … Thomas, F. (2016). The guardians of inherited oncogenic vulnerabilities. Evolution, 70, 1–6. 26519218 10.1111/evo.12809

[eva12444-bib-0015] Beatty, G. L. , Chiorean, E. G. , Fishman, M. P. , Saboury, B. , Teitelbaum, U. R. , Sun, W. , … Vonderheide, R. H. (2011). D40 agonists alter tumor stroma and show efficacy against pancreatic carcinoma in mice and humans. Science, 331, 1612–1616. 21436454 10.1126/science.1198443 PMC3406187

[eva12444-bib-0016] Ben‐David, U. , & Benvenisty, N. (2011). The tumorigenicity of human embryonic and induced pluripotent stem cells. Nature Reviews Cancer, 11, 268–277. 21390058 10.1038/nrc3034

[eva12444-bib-0017] Besedovsky, L. , Lange, T. , & Born, J. (2012). Sleep and immune function. Pflügers Archiv ‐ European Journal of Physiology, 463, 121–137. 22071480 10.1007/s00424-011-1044-0 PMC3256323

[eva12444-bib-0018] Biddle, A. , Liang, X. , Gammon, L. , Fazil, B. , Harper, L. J. , Emich, H. , … Mackenzie, I. C. (2011). Cancer stem cells in squamous cell carcinoma switch between two distinct phenotypes that are preferentially migratory or proliferative. Cancer Research, 71, 5317–5326. 21685475 10.1158/0008-5472.CAN-11-1059

[eva12444-bib-0019] Boddy, A. M. , Kokko, H. , Breden, F. , Wilkinson, G. S. , & Aktipis, C. A. (2015). Cancer susceptibility and reproductive trade‐offs: A model of the evolution of cancer defences. Philosophical Transactions of the Royal Society of London. Series B, Biological sciences, 370, 20140220. 26056364 10.1098/rstb.2014.0220 PMC4581025

[eva12444-bib-0020] Cairns, J. (1975). The cancer problem. Scientific American, 233(64–72), 77–78. 10.1038/scientificamerican1175-64 1188342

[eva12444-bib-0021] Campisi, J. (2002). Cancer and aging: Yin, yang, and p53. Science of Aging Knowledge Environment : SAGE KE, 2002, pe1. 14602974 10.1126/sageke.2002.1.pe1

[eva12444-bib-0022] Campisi, J. (2003). Cancer and ageing: Rival demons? Nature Reviews Cancer, 3, 339–349. 12724732 10.1038/nrc1073

[eva12444-bib-0023] Caulin, A. F. , & Maley, C. C. (2011). Peto's Paradox : Evolution's prescription for Cancer Prevention. Trends in Ecology & Evolution, 26, 175–182. 21296451 10.1016/j.tree.2011.01.002 PMC3060950

[eva12444-bib-0024] Chen, J. , Gong, T.‐T. , & Wu, Q.‐J. (2016). Parity and gastric cancer risk: A systematic review and dose‐response meta‐analysis of prospective cohort studies. Scientific Reports, 6, 18766. 26727146 10.1038/srep18766 PMC4698715

[eva12444-bib-0025] Chen, J. , Sprouffske, K. , Huang, Q. , & Maley, C. C . (2011). Solving the Puzzle of Metastasis : The Evolution of Cell Migration in Neoplasms. 6. 10.1371/journal.pone.0017933 PMC3083389 21556134

[eva12444-bib-0502] Crespi, B. , & Summers, K. (2005). Evolutionary biology of cancer. Trends in Ecology and Evolution, 20(10), 545–552. 16701433 10.1016/j.tree.2005.07.007

[eva12444-bib-0026] Daenen, L. G. M. , Roodhart, J. M. L. , van Amersfoort, M. , Dehnad, M. , Roessingh, W. , Ulfman, L. H. , … Voest, E. E. (2011). Chemotherapy enhances metastasis formation via VEGFR‐1‐expressing endothelial cells. Cancer Research, 71, 6976–6985. 21975929 10.1158/0008-5472.CAN-11-0627

[eva12444-bib-0027] Daoust, S. P. , Fahrig, L. , Martin, A. E. , & Thomas, F. (2013). From forest and agro‐ecosystems to the microecosystems of the human body: What can landscape ecology tell us about tumor growth, metastasis, and treatment options? Evolutionary Applications, 6, 82–91. 23396712 10.1111/eva.12031 PMC3567473

[eva12444-bib-0028] Davis, F. M. , Stewart, T. A. , Thompson, E. W. , & Monteith, G. R. (2014). Targeting EMT in cancer: Opportunities for pharmacological intervention. Trends in Pharmacological Sciences, 35, 479–488. 25042456 10.1016/j.tips.2014.06.006

[eva12444-bib-0029] De Pillis, L. G. , Gu, W. , & Radunskaya, A. E. (2006). Mixed immunotherapy and chemotherapy of tumors: Modeling, applications and biological interpretations. Journal of Theoretical Biology, 238, 841–862. 16153659 10.1016/j.jtbi.2005.06.037

[eva12444-bib-0030] DeBerardinis, R. J. , Lum, J. J. , Hatzivassiliou, G. , & Thompson, C. B. (2008). The biology of cancer: Metabolic reprogramming fuels cell growth and proliferation. Cell Metabolism, 7, 11–20. 18177721 10.1016/j.cmet.2007.10.002

[eva12444-bib-0031] DeGregori, J. (2011). Evolved tumor suppression: Why are we so good at not getting cancer? Cancer Research, 71, 3739–3744. 21610109 10.1158/0008-5472.CAN-11-0342 PMC3677553

[eva12444-bib-0032] DeWitt, T. J. , Sih, A. , & Wilson, D. S. (1998). Cost and limits of phenotypic plasticity. Trends in Ecology & Evolution, 12, 77–81. 10.1016/s0169-5347(97)01274-3 21238209

[eva12444-bib-0033] Donehower, L. A. (2002). Does p53 affect organismal aging? Journal of Cellular Physiology, 192, 23–33. 12115733 10.1002/jcp.10104

[eva12444-bib-0034] Ducasse, H. , Arnal, A. , Vittecoq, M. , Daoust, S. P. , Ujvari, B. , Jacqueline, C. , … Thomas, F. (2015). Cancer: An emergent property of disturbed resource‐rich environments? Ecology meets personalized medicine. Evolutionary Applications, 8, 527–540. 26136819 10.1111/eva.12232 PMC4479509

[eva12444-bib-0035] Easton, D. F. , Ford, D. , & Bishop, D. T. (1995). Breast and ovarian cancer incidence in BRCA1‐mutation carriers. Breast Cancer Linkage Consortium. American Journal of Human Genetics, 56, 265–271. 7825587 PMC1801337

[eva12444-bib-0036] Enriquez‐Navas, P. M. , Kam, Y. , Das, T. , Hassan, S. , Silva, A. , Foroutan, P. , … Gatenby, R. A. (2016). Exploiting evolutionary principles to prolong tumor control in preclinical models of breast cancer. Science Translational Medicine, 8, 1–20. 10.1126/scitranslmed.aad7842 PMC4962860 26912903

[eva12444-bib-0037] Fahrig, L. (2007). Non‐optimal animal movement in human‐altered landscapes. Functional Ecology, 21, 1003–1015.

[eva12444-bib-0503] Fernandez, A. A. , & Bowser, P. R. (2010). Selection for a dominant oncogene and large male size as a risk factor for melanoma in the Xiphophorus animal model. Molecular Ecology, 19(15), 3114–3123. 20618898 10.1111/j.1365-294X.2010.04738.x PMC2911510

[eva12444-bib-0504] Fernandez, A. A. , & Morris, M. R. (2008). Mate choice for more melanin as a mechanism to maintain a functional oncogene. Proceedings of the National Academy of Sciences of the United States of America, 105(36), 13503–13507. 18757731 10.1073/pnas.0803851105 PMC2533219

[eva12444-bib-0038] Finlay, C. M. , Walsh, K. P. , & Mills, K. H. G. (2014). Induction of regulatory cells by helminth parasites: Exploitation for the treatment of inflammatory diseases. Immunological Reviews, 259, 206–230. 24712468 10.1111/imr.12164

[eva12444-bib-0039] Fletcher, J. I. , Haber, M. , Henderson, M. J. , & Norris, M. D. (2010). ABC transporters in cancer: More than just drug efflux pumps. Nature Reviews Cancer, 10, 147–156. 20075923 10.1038/nrc2789

[eva12444-bib-0040] Forbes, M. R. L. (1993). Parasitism and host reproductive effort. Oikos, 67, 444–450.

[eva12444-bib-0041] Frank, S. A . (2007). Dynamics of Cancer. Princeton University Press. 20821846

[eva12444-bib-0042] García‐Cao, I. , García‐Cao, M. , Martín‐Caballero, J. , Criado, L. M. , Klatt, P. , Flores, J. M. , … Serrano, M. (2002). “Super p53” mice exhibit enhanced DNA damage response, are tumor resistant and age normally. EMBO Journal, 21, 6225–6235. 12426394 10.1093/emboj/cdf595 PMC137187

[eva12444-bib-0043] Gatenby, R. A. , Brown, J. , & Vincent, T . (2009). Lessons from Applied Ecology : Cancer Control Using an Evolutionary Double Bind. 7499–7502. 10.1158/0008-5472.CAN-09-1354 19752088

[eva12444-bib-0044] Gatenby, R. A. , Silva, A. S. , Gillies, R. J. , & Frieden, B. R. (2009). Adaptive Therapy. Cancer Research, 69, 4894–4903. 19487300 10.1158/0008-5472.CAN-08-3658 PMC3728826

[eva12444-bib-0045] Goss, P. E. , Ingle, J. N. , Alès‐Martinez, J. E. , Cheung, A. M. , Chlebowski, R. T. , Wactawski‐Wende, J. , … Richardson, H. (2011). Exemestane for breast‐cancer prevention in postmenopausal women. The New England journal of medicine, 354, 2531–2541. 10.1056/NEJMoa1103507 21639806

[eva12444-bib-0046] Gottesman, M. M. (2002). Mechanisms of cancer drug resistance. Annual Review of Medicine, 53, 615–627. 10.1146/annurev.med.53.082901.103929 11818492

[eva12444-bib-0047] Haabeth, O. A. W. , Lorvik, K. B. , Hammarström, C. , Donaldson, I. M. , Haraldsen, G. , Bogen, B. , & Corthay, A. (2011). Inflammation driven by tumour‐specific Th1 cells protects against B‐cell cancer. Nature Communications, 2, 240. 10.1038/ncomms1239 PMC3072106 21407206

[eva12444-bib-0048] Hamede, R. K. , Bashford, J. , McCallum, H. , & Jones, M. (2009). Contact networks in a wild Tasmanian devil (Sarcophilus harrisii) population: Using social network analysis to reveal seasonal variability in social behaviour and its implications for transmission of devil facial tumour disease. Ecology Letters, 12, 1147–1157. 19694783 10.1111/j.1461-0248.2009.01370.x

[eva12444-bib-0049] Harnett, W. , & Harnett, M. M. (2010). Helminth‐derived immunomodulators: Can understanding the worm produce the pill? Nature Reviews. Immunology, 10, 278–284. 10.1038/nri2730 20224568

[eva12444-bib-0050] Harshman, L. G. , & Zera, A. J. (2007). The cost of reproduction: The devil in the details. Trends in Ecology & Evolution, 22, 80–86. 17056152 10.1016/j.tree.2006.10.008

[eva12444-bib-0051] Hawkins, B. A. (2004). Are we making progress toward understanding the global diversity gradient? Basic and Applied Ecology, 5, 1–3.

[eva12444-bib-0052] Hochberg, M. E. , Michalakis, Y. , & de Meeus, T. (1992). Parasitism as a constraint on the rate of life‐history evolution. Journal of Evolutionary Biology, 5, 491–504.

[eva12444-bib-0053] Hochberg, M. E. , Thomas, F. , Assenat, E. , & Hibner, U. (2013). Preventive evolutionary medicine of cancers. Evolutionary Applications, 6, 134–143. 23396860 10.1111/eva.12033 PMC3567478

[eva12444-bib-0054] Huncharek, M. , Caubet, J. F. , & McGarry, R. (2001). Single‐agent DTIC versus combination chemotherapy with or without immunotherapy in metastatic melanoma: A meta‐analysis of 3273 patients from 20 randomized trials. Melanoma Research, 11, 75–81. 11254118 10.1097/00008390-200102000-00009

[eva12444-bib-0056] Ingels, A. , Sanchez Salas, R. E. , Ravery, V. , Fromont‐Hankard, G. , Validire, P. , Patard, J.‐J. , … Cathelineau, X. (2014). T‐helper 1 immunoreaction influences survival in muscle‐invasive bladder cancer: Proof of concept. Ecancermedicalscience, 8, 486. 25525464 10.3332/ecancer.2014.486 PMC4263522

[eva12444-bib-0057] Kam, Y. , Das, T. , Tian, H. , Foroutan, P. , Ruiz, E. , Martinez, G. , … Gatenby, R. A. (2011). Sweat but no gain: inhibiting proliferation of multidrug resistant cancer cells with “Ersatzdroges.”. International Journal of Cancer, 2, 722–731. 10.1002/ijc.29158 PMC4262548 25156304

[eva12444-bib-0058] Key, T. , Appleby, P. , Barnes, I. , Reeaves, G. (2002). Endogenous sex hormones and breast cancer in postmenopausal women : Reanalysis of nine prospective studies. Journal of the National Cancer Institute, 94, 606–616. 11959894 10.1093/jnci/94.8.606

[eva12444-bib-0059] Khan, D. , & Ansar Ahmed, S. (2016). The immune system is a natural target for estrogen action: Opposing effects of estrogen in two prototypical autoimmune diseases. Frontiers in Immunology, 6, 1–8. 10.3389/fimmu.2015.00635 PMC4701921 26779182

[eva12444-bib-0060] Khanna, K. K. , & Jackson, S. P. (2001). DNA double‐strand breaks: Signaling, repair and the cancer connection. Nature Genetics, 27, 247–254. 11242102 10.1038/85798

[eva12444-bib-0061] Kidd, P. (2003). Th1/Th2 balance: The hypothesis, its limitations, and implications for health and disease. Alternative Medicine Review : A Journal of Clinical Therapeutic, 8, 223–246. 12946237

[eva12444-bib-0062] Lee, H. , Silva, A. , Li, Y. , & Slifker, M. (2011). Evolution of tumor invasiveness: The adaptive tumor microenvironment landscape model. Cancer Research, 71, 6327–6337. 21859828 10.1158/0008-5472.CAN-11-0304 PMC3197231

[eva12444-bib-0063] Lemaitre, J. , Berger, V. , Bonenfant, C. , Douhard, M. , Gamelon, M. , Plard, F. , & Gaillard, J. (2015). Early‐late life trade‐offs and the evolution of ageing in the wild. Proceedings of the Royal Society B‐Biological Sciences, 282, 20150209. 10.1098/rspb.2015.0209 PMC4426628 25833848

[eva12444-bib-0064] León‐Cabrera, S. , Callejas, B. E. , Ledesma‐Soto, Y. , Coronel, J. , Pérez‐Plasencia, C. , Gutiérrez‐Cirlos, E. B. , … Terrazas, L. I. (2014). Extraintestinal helminth infection reduces the development of colitis‐associated tumorigenesis. International journal of biological sciences, 10, 948–956. 25210492 10.7150/ijbs.9033 PMC4159685

[eva12444-bib-0065] Lima, S. L. , Rattenborg, N. C. , Lesku, J. A. , & Amlaner, C. J. (2005). Sleeping under the risk of predation. Animal Behaviour, 70, 723–736.

[eva12444-bib-0066] Lippitz, B. E. (2013). Cytokine patterns in patients with cancer: A systematic review. The Lancet. Oncology, 14, e218–e228. 23639322 10.1016/S1470-2045(12)70582-X

[eva12444-bib-0067] Lynch, M. , Field, M. C. , Goodson, H. V. , Malik, H. S. , Pereira‐Leal, J. B. , Roos, D. S. , … Sazer, S. (2014). Evolutionary cell biology: Two origins, one objective. Proceedings of the National Academy of Sciences of the United States of America, 111, 16990–16994. 25404324 10.1073/pnas.1415861111 PMC4260604

[eva12444-bib-0068] Lynch, K. S. , Rand, A. S. , Ryan, M. J. , & Wilczynski, W. (2005). Plasticity in female mate choice associated with changing reproductive states. Animal Behaviour, 69, 689–699.

[eva12444-bib-0069] Machiels, J. H. , Reilly, R. T. , Emens, L. A. , Vaccines, W. , Tolerized, H. , Ercolini, A. M. , … Jaffee, E. M. (2001). Cyclophosphamide, doxorubicin, and paclitaxel enhance the antitumor immune response of granulocyte/macrophage‐colony stimulating factor‐secreting whole‐cell vaccines in HER‐2/neu tolerized mice cyclophosphamide, doxorubicin, and paclitaxel enhance. Cancer Research, 61, 3689–3697. 11325840

[eva12444-bib-0070] Makkouk, A. , & Weiner, G. J. (2015). Cancer immunotherapy and breaking immune tolerance: New approaches to an old challenge. Cancer Research, 75, 5–10. 25524899 10.1158/0008-5472.CAN-14-2538 PMC4286422

[eva12444-bib-0071] Maley, C. C. , Reid, B. J. , & Forrest, S. (2004). Cancer prevention strategies that address the evolutionary dynamics of neoplastic cells : Simulating benign cell boosters and selection for chemosensitivity. Cancer epidemiology, biomarkers & prevention : a publication of the American Association for Cancer Research, cosponsored by the American Society of Preventive Oncology, 13, 1375–1385. 15298961

[eva12444-bib-0072] Mapara, M. Y. , & Sykes, M. (2004). Tolerance and cancer: Mechanisms of tumor evasion and strategies for breaking tolerance. Journal of Clinical Oncology, 22, 1136–1151. 15020616 10.1200/JCO.2004.10.041

[eva12444-bib-0073] Marusyk, A. , & Polyak, K. (2010). Tumor heterogeneity: Causes and consequences. Biochimica et Biophysica Acta, 1805, 105–117. 19931353 10.1016/j.bbcan.2009.11.002 PMC2814927

[eva12444-bib-0074] Marusyk, A. , Tabassum, D. P. , Altrock, P. M. , Almendro, V. , Michor, F. , & Polyak, K. (2014). Non‐cell‐autonomous driving of tumour growth supports sub‐clonal heterogeneity. Nature, 514, 54–58. 25079331 10.1038/nature13556 PMC4184961

[eva12444-bib-0075] Maynard‐Smith, J. , & Szathmary, E . (1997). The Major Transitions in Evolution. 10.1006/jtbi.1996.0389 9299299

[eva12444-bib-0076] Meijers‐Heijboer, H. , van Geel, B. , van Putten, W. L. , Henzen‐Logmans, S. C. , Seynaeve, C. , Menke‐Pluymers, M. B. , … Klijn, J. G. (2001). Breast cancer after prophylactic bilateral mastectomy in women with a BRCA1 or BRCA2 mutation. The New England Journal of Medicine, 345, 159–64. 11463009 10.1056/NEJM200107193450301

[eva12444-bib-0077] Merlo, L. M. F. , Pepper, J. W. , Reid, B. J. , & Maley, C. C. (2006). Cancer as an evolutionary and ecological process. Nature Reviews Cancer, 6, 924–935. 17109012 10.1038/nrc2013

[eva12444-bib-0078] Michalakis, Y. , & Hochberg, M. E. (1994). Parasitic effects on host life‐history traits: A review of recent studies. Parasite : journal de la Société Française de Parasitologie, 1, 291–294. 10.1051/parasite/1994014291 9140497

[eva12444-bib-0079] Michod, R . (2000). Darwinian dynamics: evolutionary transitions in fitness and individuality.

[eva12444-bib-0080] Michod, R. , & Roze, D . (2001). Cooperation and conflict in the evolution of multicellularity. Heredity (Edinb). 10.1046/j.1365-2540.2001.00808.x 11298810

[eva12444-bib-0081] Møller, A. P. , & Mousseau, T. A. (2015). Strong effects of ionizing radiation from Chernobyl on mutation rates. Scientific Reports, 5, 8363. 25666381 10.1038/srep08363 PMC4322348

[eva12444-bib-0082] Moret, Y. , & Schmid‐Hempel, P. (2000). Survival for immunity : The price of immune system activation for bumblebee workers. Science, 290, 1166–1169. 11073456 10.1126/science.290.5494.1166

[eva12444-bib-0083] van der Most, P. J. , de Jong, B. , Parmentier, H. K. , & Verhulst, S. (2011). Trade‐off between growth and immune function: A meta‐analysis of selection experiments. Functional Ecology, 25, 74–80.

[eva12444-bib-0084] Murchison, E. (2009). Clonally transmissible cancers in dogs and Tasmanian devils. Oncogene, 27, S19–S30. 10.1038/onc.2009.350 19956175

[eva12444-bib-0085] Nagy, J. D. , Victor, E. M. , & Cropper, J. H. (2007). Why don't all whales have cancer? A novel hypothesis resolving Peto's paradox. Integrative and Comparative Biology, 47, 317–328. 21672841 10.1093/icb/icm062

[eva12444-bib-0086] Nesse, R. M. , & Williams, G. C . (1996).Why We Get Sick: The New Science of Darwinian Medicine.

[eva12444-bib-0087] North, A. , Cornell, S. , & Ovaskainen, O. (2011). Evolutionary responses of dispersal distance to landscape structure and habitat loss. Evolution, 65, 1739–1751. 21644960 10.1111/j.1558-5646.2011.01254.x

[eva12444-bib-0088] Nowell, P. C. (1976). The clonal evolution of tumor cell populations. Science, 194, 23–28. 959840 10.1126/science.959840

[eva12444-bib-0089] Nunney, L. (2013). The real war on cancer: The evolutionary dynamics of cancer suppression. Evolutionary Applications, 6, 11–19. 23396311 10.1111/eva.12018 PMC3567467

[eva12444-bib-0090] O'Byrne, K. J. , & Dalgleish, A. G. (2000). Evolution, immune response, and cancer. Lancet, 356, 1033–1034. 10.1016/S0140-6736(05)72656-8 11041429

[eva12444-bib-0091] Oikonomopoulou, K. , Brinc, D. , Kyriacou, K. , & Diamandis, E. P. (2013). Infection and cancer: Revaluation of the hygiene hypothesis. Clinical Cancer Research, 19, 2834–2841. 23536438 10.1158/1078-0432.CCR-12-3661

[eva12444-bib-0092] Oli, M. K. (2004). The fast–slow continuum and mammalian life‐history patterns: An empirical evaluation. Basic and Applied Ecology, 5, 449–463.

[eva12444-bib-0093] Page, D. B. , Postow, M. A. , Callahan, M. K. , & Wolchok, J. D. (2012). Checkpoint modulation in melanoma : An update on Ipilimumab and future directions. Current Oncology Reports, 100, 130–134. 10.1007/s11912-013-0337-1 PMC3799873 23933888

[eva12444-bib-0094] Pearse, A‐M. , & Swift, K. (2006). Allograft theory: Transmission of devil facial‐tumour disease. Nature, 439, 549. 16452970 10.1038/439549a

[eva12444-bib-0095] Peto, R. , Roe, F. J. , Lee, P. N. , Levy, L. , & Clack, J. (1975). Cancer and ageing in mice and men. British Journal of Cancer, 32, 411–426. 1212409 10.1038/bjc.1975.242 PMC2024769

[eva12444-bib-0096] Pitnick, S. , Jones, K. E. , & Wilkinson, G. S . (2006). Mating system and brain size in bats. 719–724. 10.1098/rspb.2005.3367 PMC1560082 16608692

[eva12444-bib-0097] Poitrineau, K. , Brown, S. P. , & Hochberg, M. E. (2003). Defence against multiple enemies. Journal of Evolutionary Biology, 16, 1319–1327. 14640423 10.1046/j.1420-9101.2003.00585.x

[eva12444-bib-0098] Promislow, D. E. L. , & Harvey, P. H. (1990). Living fast and dying young: A comparative analysis of life‐history variation among mammals. Journal of Zoology, 220, 417–437.

[eva12444-bib-0099] Pye, R. , Pemberton, D. , Tovar, C. , Tubio, J. M. C. , Dun, K. A. , Fox, S. , … Woods, G. (2016). A second transmissible cancer in Tasmanian devils. PNAS, 113, 374–379. 26711993 10.1073/pnas.1519691113 PMC4720317

[eva12444-bib-0100] Råberg, L. , Graham, A. L. , & Read, A. F. (2009). Decomposing health: Tolerance and resistance to parasites in animals. Philosophical Transactions of the Royal Society of London. Series B, Biological sciences, 364, 37–49. 18926971 10.1098/rstb.2008.0184 PMC2666700

[eva12444-bib-0101] Read, A. F. , Graham, A. L. , & Råberg, L. (2008). Animal defenses against infectious agents: Is damage control more important than pathogen control. PLoS Biology, 6, e4. 10.1371/journal.pbio.1000004 PMC2605932 19222305

[eva12444-bib-0102] Read, A. F. , & Harvey, P. H. (1989). Life history differences among the eutherian radiations. Journal of Zoology, 219, 329–353.

[eva12444-bib-0103] Restifo, N. P. , Smyth, M. J. , & Snyder, A. (2016). Acquired resistance to immunotherapy and future challenges. Nature Reviews Cancer, 16, 121–126. 26822578 10.1038/nrc.2016.2 PMC6330026

[eva12444-bib-0104] Ricklefs, R. E. (1992). Embryonic development period and the prevalence of avian blood parasites. Proceedings of the National Academy of Sciences, 89, 4722–4725. 10.1073/pnas.89.10.4722 PMC49155 1584808

[eva12444-bib-0105] Roche, B. , & Thomas, F . (2016). Third International Biannual Evolution and Cancer Conference (Evolutionary Tradeoffs and Clinical Consequences). Meeting report. San Francisco, CA, USA. 10–13 December 2015. Evol. Appl.

[eva12444-bib-0106] Roddam, A. W. , Allen, N. E. , Appleby, P. , & Key, T. J. (2008). Endogenous sex hormones and prostate cancer: A collaborative analysis of 18 prospective studies. Journal of the National Cancer Institute, 100, 170–183. 18230794 10.1093/jnci/djm323 PMC6126902

[eva12444-bib-0107] Roff, D. A . (1993). Evolution Of Life Histories: Theory and Analysis.

[eva12444-bib-0109] Rothwell, P. M. , Wilson, M. , Price, J. F. , Belch, J. F. , Meade, T. W. , & Mehta, Z. (2012). Effect of daily aspirin on risk of cancer metastasis: A study of incident cancers during randomised controlled trials. Lancet, 379, 1591–1601. 22440947 10.1016/S0140-6736(12)60209-8

[eva12444-bib-0110] Rozhok, A. I. , & DeGregori, J. (2015). Toward an evolutionary model of cancer: Considering the mechanisms that govern the fate of somatic mutations. Proceedings of the National Academy of Sciences, 112, 8914–8921. 10.1073/pnas.1501713112 PMC4517250 26195756

[eva12444-bib-0111] Samson, D. R. , & Nunn, C. L. (2015). Sleep Intensity and the Evolution of Human Cognition. Evolutionary Anthropology. 24, 225–237. 26662946 10.1002/evan.21464

[eva12444-bib-0112] Sapolsky, R. M. (2016). Social Status and Health in Humans and Other Animals Social Status and Health in Humans and Other Animals. Annual Review of Anthropology, 33, 393–418.

[eva12444-bib-0113] Senar, J. C. , Møller, A. P. , Ruiz, I. , Negro, J. J. , Broggi, J. , & Hohtola, E. (2010). Specific appetite for carotenoids in a colorful bird. PLoS ONE, 5, e10716. 20502717 10.1371/journal.pone.0010716 PMC2873299

[eva12444-bib-0114] Sheldon, B. C. , & Verhulst, S. (1996). Ecological immunology : Costly parasite defences and trade‐offs in evolutionary ecology. Trends in Ecology & Evolution, 5347, 317–321. 10.1016/0169-5347(96)10039-2 21237861

[eva12444-bib-0115] Silva, A. S. , Kam, Y. , Khin, Z. P. , Minton, S. E. , Gillies, R. J. , & Gatenby, R. A. (2012). Evolutionary approaches to prolong progression‐free survival in breast cancer. Cancer Research, 72, 6362–6370. 23066036 10.1158/0008-5472.CAN-12-2235 PMC3525750

[eva12444-bib-0116] Smith, K. R. , Hanson, H. A. , Mineau, G. P. , & Buys, S. S. (2012). Effects of BRCA1 and BRCA2 mutations on female fertility. Proceedings of the Royal Society B‐Biological Sciences, 279, 1389–1395. 10.1098/rspb.2011.1697 PMC3282366 21993507

[eva12444-bib-0117] Söderlund‐Strand, A. , Uhnoo, I. , & Dillner, J. (2014). Change in population prevalences of human papillomavirus after initiation of vaccination: The high‐throughput HPV monitoring study. Cancer Epidemiology, Biomarkers & Prevention : A Publication Of The American Association for Cancer Research, Cosponsored by the American Society Of Preventive Oncology, 23, 2757–2765. 10.1158/1055-9965.EPI-14-0687 25380734

[eva12444-bib-0118] Sorci, G. , Boulinier, T. , Gauthier‐Clerc, M. , & Faivre, B . (2008). The evolutionary ecology of the immune response. Oxford University Press.

[eva12444-bib-0119] Stearns, S. C . (1989). Trade‐offs in life‐history Evolution.

[eva12444-bib-0120] Stearns, S . (1992). The evolution of life histories.

[eva12444-bib-0121] Summers, K. , & Crespi, B. (2008). The androgen receptor and prostate cancer: A role for sexual selection and sexual conflict?. Medical Hypotheses, 70, 435–443. 17656031 10.1016/j.mehy.2007.04.044

[eva12444-bib-0122] Thomas, F. , Fisher, D. , Fort, P. , Marie, J.‐P. , Daoust, S. , Roche, B. , … Hochberg, M. E. (2013). Applying ecological and evolutionary theory to cancer: A long and winding road. Evolutionary Applications, 6, 1–10. 23397042 10.1111/eva.12021 PMC3567465

[eva12444-bib-0123] Thomas, F. , Guégan, J.‐F. , & Renaud, F . (2009). Ecology and Evolution of Parasitism. Oxford Uni.

[eva12444-bib-0124] Thomas, F. , Nesse, R. M. , Gatenby, R. , Gidoin, C. , Renaud, F. , Roche, B. , & Ujvari, B. (2016). Evolutionary ecology of organs: A missing link in cancer development? Trends in Cancer, 2, 409–415. 10.1016/j.trecan.2016.06.009 28741494

[eva12444-bib-0125] Thomas, F. , Roche, B. , & Ujvari, B . (2016). Intrinsic versus extrinsic cancer risks: The debate continues. Trends in Cancer. 2, 68–69. 10.1016/j.trecan.2016.01.004 28741552

[eva12444-bib-0126] Thompson, C. L. , & Li, L. (2013). Association of sleep duration and breast cancer oncotype DX recurrence score. Breast Cancer Research and Treatment, 134, 1291–1295. 10.1007/s10549-012-2144-z PMC3409927 22752291

[eva12444-bib-0127] Tissot, T. , Arnal, A. , Jacqueline, C. , Poulin, R. , Lefèvre, T. , Mery, F. , … Thomas, F. (2016). Host manipulation by cancer cells: Expectations, facts, and therapeutic implications. BioEssays, 38, 276–285. 26849295 10.1002/bies.201500163

[eva12444-bib-0128] Tomasetti, C. , & Vogelstein, B. (2014). Variation in cancer risk among tissues can be explained by the number of stem cell divisions. Science, 347, 78–81. 10.1126/science.1260825 PMC4446723 25554788

[eva12444-bib-0129] Trumble, B. C. , Blackwell, A. D. , Stieglitz, J. , Emery, M. , Ivan, T. , Suarez, M. , … Gurven, M . (2016). Associations between male testosterone and immune function in a pathogenically stressed forager‐horticultural population. 1–12. 10.1002/ajpa.23054 PMC5075254 27465811

[eva12444-bib-0130] Tyner, S. D. , Venkatachalam, S. , Choi, J. , Jones, S. , Ghebranious, N. , Igelmann, H. , … Donehower, L. A. (2002). p53 mutant mice that display early ageing‐associated phenotypes. Nature, 415, 45–53. 11780111 10.1038/415045a

[eva12444-bib-0131] Ujvari, B. , Beckmann, C. , Biro, P. A. , Arnal, A. , Tasiemski, A. , Massol, F. , … Thomas, F. (2016). Cancer and life‐history traits: Lessons from host‐parasite interactions. Parasitology, 143, 533–541. 26887797 10.1017/S0031182016000147

[eva12444-bib-0132] Valle, B. L. , D'Souza, T. , Becker, K. G. , Wood, W. H. , Zhang, Y. , Wersto, R. P. , & Morin, P. J. (2013). Drugs, non‐steroidal anti‐inflammatory decrease E2F1 expression, & growth, inhibit cell in cells, ovarian cancer. PLoS ONE, 8, e61836. 23637916 10.1371/journal.pone.0061836 PMC3634839

[eva12444-bib-0133] Vittecoq, M. , Ducasse, H. , Arnal, A. , Møller, A. P. , Ujvari, B. , Jacqueline, C. B. , … Thomas, F. (2015). Animal behaviour and cancer. Animal Behaviour, 101, 19–26.

[eva12444-bib-0134] Walker, B. , Figgs, L. W. , & Zahm, S. H. (1995). Differences in cancer incidence, mortality, and survival between African Americans and Whites. Environmental Health Perspectives, 103, 275–281. 8741798 10.1289/ehp.95103s8275 PMC1518956

[eva12444-bib-0135] Wheeler, C. J. , Das, A. , Liu, G. , Yu, J. S. , & Black, K. L. (2004). Clinical responsiveness of glioblastoma multiforme to chemotherapy after vaccination. Clinical Cancer Research: an Official Journal of the American Association for Cancer Research, 10, 5316–5326. 15328167 10.1158/1078-0432.CCR-04-0497

[eva12444-bib-0136] Williams, G. C. , & Nesse, R. M. (1991). The dawn of Darwinian medicine. Quarterly Review of Biology, 66, 1–22. 2052670 10.1086/417048

[eva12444-bib-0137] Wolfe, N. D. , Dunavan, C. P. , & Diamond, J. (2007). Origins of major human infectious diseases. Nature, 447, 279–283. 17507975 10.1038/nature05775 PMC7095142

[eva12444-bib-0138] Wu, Q.‐J. , Li, Y.‐Y. , Tu, C. , Zhu, J. , Qian, K.‐Q. , Feng, T.‐B. , … Ma, X.‐X. (2015). Parity and endometrial cancer risk: A meta‐analysis of epidemiological studies. Scientific Reports, 5, 14243. 26373341 10.1038/srep14243 PMC4642705

[eva12444-bib-0139] Yaddanapudi, K. , Mitchell, R. A. , Putty, K. , Willer, S. , Sharma, R. K. , Yan, J. , … Eaton, J. W. (2012). Vaccination with embryonic stem cells protects against lung cancer: Is a broad‐spectrum prophylactic vaccine against cancer possible? PLoS ONE, 7, e42289. 22860107 10.1371/journal.pone.0042289 PMC3409174

[eva12444-bib-0140] Zacharia, B. E. , Zacharia, B. , & Sherman, P. (2003). Atopy, helminths, and cancer. Medical Hypotheses, 60, 1–5. 12450762 10.1016/s0306-9877(02)00217-7

